# Executive dysfunction as a possible mediator for the association between excessive screen time and problematic behaviors in preschoolers

**DOI:** 10.1371/journal.pone.0298189

**Published:** 2024-04-04

**Authors:** Narueporn Likhitweerawong, Nonglak Boonchooduang, Jiraporn Khorana, Phichayut Phinyo, Jayanton Patumanond, Orawan Louthrenoo

**Affiliations:** 1 Department of Pediatrics, Division of Growth and Development, Faculty of Medicine, Chiang Mai University, Chiang Mai, Thailand; 2 Department of Surgery, Division of Pediatric Surgery, Faculty of Medicine, Chiang Mai University, Chiang Mai, Thailand; 3 Center of Clinical Epidemiology and Clinical Statistics, Faculty of Medicine, Chiang Mai University, Chiang Mai, Thailand; 4 Clinical Surgical Research Center, Department of Surgery, Faculty of Medicine, Chiang Mai University, Chiang Mai, Thailand; 5 Department of Family Medicine, Faculty of Medicine, Chiang Mai University, Chiang Mai, Thailand; University of Montenegro, MONTENEGRO

## Abstract

Excessive screen time in children is a growing concern for parents and healthcare providers worldwide because it frequently leads to behavioral problems. Although executive dysfunction is proposed to be one of the contributing factors to maladaptive behaviors, little is known about the link between screen time and behaviors. This study aimed to identify whether executive dysfunction contributes to the negative behaviors of children exposed to excessive screen time. A cross-sectional study was conducted on preschool-aged children from public and private schools in Chiang Mai, Thailand. The parents/guardians of each child completed the questionnaires regarding clinical characteristics and screen time use, the Behavior Rating Inventory for Executive Function-Preschool (BRIEF-P), and the Strengths and Difficulties Questionnaire (SDQ). Children with more than one hour of media exposure per day were considered to have excessive screen time. Multivariable Gaussian regression was analyzed to compare the BRIEF-P and SDQ scores between the excessive and appropriate screen time groups. Causal mediation analysis was performed to examine the effects of total screen time on increasing behavioral problems with executive functioning as a mediator. A total of 1,126 preschoolers were included in the analyses. After controlling for age, sex, socioeconomic status, and maternal education, the excessive screen time group had significantly higher BRIEF-P global executive composite score than the appropriate screen time group (mean difference of global executive composite score = 1.49, 95% CI [0.12, 2.86], and *p* = 0.033). Concurrently, there were significant differences in externalizing behavior subscales and SDQ total difficulties scores between the excessive and appropriate screen time groups (mean difference of total difficulties score = 0.90, 95% CI [0.29, 1.50], and *p* = 0.004). A significant average causal mediation effect (ACME) of screen time on behavioral problems mediated through executive functioning was β = 0.28, 95% CI [0.13–0.44], which was more than half of the total effect (54.9%, 95% CI [37.4–100%]). The current study suggests that the increase in behavioral issues in preschoolers might be partly explained by the direct effect of excessive screen time and the mediating effect of impaired executive functioning. Our results may raise concerns about the necessity to limit screen time and monitor for executive function deficits and behavioral problems in young children with high screen time.

## Introduction

Over the past decades, screen usage in children and youth has increased worldwide due to digital advancements. A steep rise in the rate of excessive media use in young children, especially during the COVID-19 pandemic, worsens the situation. Despite the American Academy of Pediatrics (AAP) recommendation regarding age-appropriate screen time [1], the average daily screen use in children aged 2–4 and 5–8 years is still 2.5 and 3 hours, respectively [2]. In Thailand, excessive screen time is also prevalent among preschoolers [3]. Apart from prolonged school closing and home confinement resulting in excessive screen time, more than half of parents perceive their child has learning benefits from the screen [2, 3]. Although some learning abilities can be improved by high-quality child-directed digital media, very young children may gain little benefit from them [4]. Using screens at this younger age is probably not beneficial and may even be harmful.

A growing body of research indicates that excessive screen time negatively impacts children [5–8]. For example, a high frequency of television watching was associated with language delay in children aged 15–48 months [5]. Screen time has increased the incidence of weight excess [6]. Longer television viewing time was related to shorter total sleep duration in young children [7, 8]. Not only does excessive screen time affect physical and developmental health, as mentioned above, but it also impacts behavioral problems [9–12]. There is an elevated risk of having negative behaviors such as emotional, conduct, peer, and hyperactivity problems in preschoolers who spend over one hour of screen time per day [9, 10]. The likelihood of displaying hyperactive behaviors increased as screen time was prolonged [11]. In addition, a comprehensive review examined the relationship between screen time and attention deficit hyperactivity disorder (ADHD)-related behaviors and found a statistically significant association [12].

Executive function (EF) is a higher-level cognitive control process involving thoughts/behavior regulations, attention, and task completion [13, 14]. It is comprised of the essential EF components, which are inhibition/inhibitory control, updating/working memory, and shifting/cognitive flexibility, and high-order EFs such as problem-solving and planning [13, 14]. Although EF continues to develop throughout late adolescence, the most significant changes are seen in the preschool ages [15], when there is significant activation of the prefrontal cortex, which regulates EF [16]. From early childhood through early adulthood, EF typically develops naturally, but environmental variables also strongly impact it [17, 18]. Notably, excessive screen exposure has been proposed as a possible environmental factor contributing to EF deficits [19]. Studies have shown that children with higher total television viewing time exhibited poorer EF compared to those with lower viewing times [20–22]. This association was also proved in the previous longitudinal study that showed a significant linear effect of increased screen time on poor EF outcomes one year later [18]. Several theoretical hypotheses attempt to explain this association. Firstly, elevated sensory inputs from the screen, particularly involving fast-paced and fantastical contents, increased reliance on attention in a bottom-up manner (stimulus-driven attention; captured attention by salient stimuli), creating challenges for effective top-down regulation (goal-directed attention; the internal focus guided by prior knowledge, goals, and plans, which enables one to maintain the concentration to the current task.) and progression of executive functioning [23–25]. Secondly, young children struggle to learn content from screens and differentiate between reality and fantasy. Exposure to screens at young ages, especially those containing age-inappropriate content, demands more cognitive processing to translate and store the novel occurrences that are unfamiliar to the child. Once these mental resources are exhausted, fewer cognitive capacities are available for subsequent executive functioning [23, 24]. Lastly, young children greatly benefit from reciprocal interaction with others for their developmental progression [26]. Screen time may replace the interaction time with caregivers and other play-related tasks, which are essential for EF development [18]. Thus, repeated excessive exposure to screens may hinder the typical cognitive processes in the developing child, potentially leading to EF problems [27].

In terms of EF and behavior issues, EF is closely related to social-emotional competencies, and it is essential for socially appropriate behaviors [28–30]. For example, EF helps individuals control impulsiveness/aggression and comply with social rules. It also facilitates processing and updating socially relevant information, enabling individuals to adapt to changing social situations in a flexible manner [29, 30]. Emotional development, including empathy and the ability to infer other’s feelings and intentions, also plays a crucial role in fostering positive social interactions [31]. Given that, children with EF deficits may encounter challenges in managing their behaviors. A meta-analysis of longitudinal studies has indicated an inverse correlation between EF and the occurrence of internalizing (e.g., depression symptoms) and externalizing (e.g., ADHD and oppositional defiant disorder symptoms) behaviors [32]. Generally, executive dysfunction has been described as one of the contributing factors to disruptive behaviors in children with neurodevelopmental disorders such as autism spectrum disorders, ADHD, and epilepsy [33, 34]. Growing evidence reveals that neurotypical children also exhibit these disruptive behaviors due to their EF deficits [35]. In addition, evidence from a meta-analysis confirms that impaired EF was linked to externalizing behavior problems, and this association is evident as early as during the preschool years [36].

It is noteworthy to investigate whether EF impairment resulting from excessive screen exposure could affect young children’s behaviors. Although a recent meta-analysis has established a significant association between excessive screen time and problematic behaviors [37], the direct/indirect mechanisms underlying this relationship still need to be clarified. There is only one study showing that some aspects of executive control difficulties due to increased screen time link to higher scores on rating ADHD symptoms in adolescents [38]. However, quantitative analyses addressing the mediating role of EF between screen time and behavioral problems in preschoolers are lacking. We hypothesized that EF was the potential mediator of excessive screen exposure on behavioral problems in preschool-aged children, the most critical EF development period. This study aimed to determine whether EF mediated such an association. A model explaining the relationship among these variables might raise awareness of the need to strictly monitor screen time, and it may help to conceptualize EF-targeted interventions to reduce challenging behaviors from inappropriate screen use in young children.

## Materials and methods

### Study design and participants

This study was a secondary analysis from the large population-based cross-sectional EF study [39]. The sample was drawn from the seven private and public schools in Chiang Mai city, Thailand. We selected an appropriate sample of schools representing middle-class socioeconomic status (SES). Children raised in the middle SES could be a good representative for studying screen exposure and behavioral problems. Since family members usually have internet access and are fairly digitally literate, children in middle SES are more likely to be exposed to screens than those with low SES. In addition, generally healthy and typically developing children in middle SES represent the majority of the pediatric population. This was evident by a large Thai survey that showed a significant proportion of the country’s population falls under the middle SES category [40]. Schools representing middle-class SES were chosen based on characteristics such as the size of the school, the number of students, and school fees. Finally, seven representative schools were obtained by using stratified sampling. The sampling process in more detail was reported elsewhere [39]. The power analysis of the sample size used in this study was performed and found to have sufficient power to detect the effect.

The child’s parents/guardians were given the general questionnaire regarding the child’s information, the parent report Behavior Rating Inventory for Executive Function-Preschool, and the parent report Strengths and Difficulties Questionnaire. One thousand five hundred and sixty children met the inclusion criteria: preschoolers four to five years of age who were studying in kindergartens one to three. The exclusion criteria included: (1) participants with genetic diseases or neurodevelopmental disorders (e.g., Down syndrome, Fragile X syndrome, intellectual disability, autism spectrum disorder, attention-deficit/hyperactivity disorder, and cerebral palsy) reported by their parents/guardians; (2) not being ethnically Thai; and (3) refusing the study participation. The study was approved by the Research Ethics Committee of the Faculty of Medicine, Chiang Mai University (approval number 051/2564). Written informed consent was obtained from the parents/guardians of the child before participating in the study. We collected the individual information in the format of identification number. Thus, the data were analyzed anonymously.

### Measures

#### Demographics and clinical characteristics

The child’s age, sex, family income, and maternal highest education level were obtained through the general questionnaire. The family income was categorized into SES followed by the Thai socioeconomic status Classification [41]. Low SES refers to a monthly family income equal to or less than 18,000 baht (1 USD = 34 THB in 2023). Middle SES refers to a monthly family income of over 18,000 to 85,000 baht. High SES refers to a monthly family income of over 85,000 baht [41]. Parenting styles were assessed using the Parenting Styles and Dimensions Questionnaire (PSDQ) Short form–Thai [42]. It evaluates the caregiver’s parenting behaviors, which are authoritative, authoritarian, and permissive, as described by Baumrind [43]. Higher authoritative parenting style scores reflect higher parental involvement and attentiveness to the child. The authoritarian style indicates parents have high expectations of their children and strict rule-following but low responsiveness, whereas the permissive style reflects high responsiveness but low demands. This tool displays good validity and reliability [42]. The total duration of sleep (including naps) and physical activity level were determined by sleep and physical activity questions, which were developed based on age-appropriate sleep and physical activity recommendations [44, 45]. These questions were validated and demonstrated acceptable reliability and validity (S1 Table).

#### Screen time

Total screen time accumulated in one day was evaluated from the question: ‘On weekdays, how much does your child spend time/exposed to the screen such as mobile phone, tablet, computer, and television throughout the day?’ The parents/guardians responded to the question using free text for minutes or hours per day. The same question was repeated for weekends. Total screen time was the average amount of time per day spent watching a screen on weekdays and weekends. According to the AAP recommendation regarding screen time, the appropriate screen time is no more than one hour per day for a child aged two to five years [1]. Therefore, the cut-off screen time of one hour per day was used to categorize the participants into two groups (excessive screen time vs. appropriate screen time groups).

#### Executive function

Executive function was assessed using the parent report Behavior Rating Inventory for Executive Function-Preschool—Thai (BRIEF-P) [46]. It was used to measure the child’s executive functioning in their everyday life as observed by their caregiver. There are 63 items with the 3-point Likert scale, including the clinical scales (i.e., inhibition, shift, emotional control, working memory, and plan/organization) and the global executive composite scale (GEC; the sum of these five clinical scales). Higher T-scores for each scale represent more difficulties in executive functioning. A T-score greater than or equal to 65 indicates clinically significant impaired executive functioning. The BRIEF-P is a well-validated standardized executive function measure with good reliability and validity [46].

#### Behavioral problems

Behavioral problems were assessed using the Strengths and Difficulties Questionnaire (SDQ) parent report–Thai [47]. The SDQ is a 25-item measure with a 3-point Likert scale that evaluates four dimensions of difficulties (i.e., hyperactivity/inattention, emotional symptoms, conduct problems, and peer relationship problems) and one dimension of strengths (prosocial behaviors). The four dimensions of negative behaviors were added together to generate a total difficulties score used in the causal mediation analysis. Higher scores on the negative behavior dimension indicate more behavioral problems, whereas higher scores on positive behavior reflect greater prosocial behavior. The Thai normative scores and cut-off points for each dimension determine normal, borderline, and clinically significant problems [48]. The original parent and teacher report SDQ has been widely used in studies and clinical practices with satisfactory psychometric properties [49]. Additionally, the SDQ Thai version shows sufficiently favorable internal reliabilities [48].

### Conceptual model and variables

To explore the mechanisms of exposure’s effects on outcome, we performed a causal mediation analysis. The total effect of exposure on the outcome was dissected into a direct effect and indirect effect, which operated through the mediator. Due to the causal mediation analysis framework [50, 51], a mediation model in this study included a mediator (executive function) in the pathway between exposure (screen time) and outcome (behavioral problems), potential covariates (age, sex, maternal education, socioeconomic status), and unmeasured mediator-outcome confounder. A conceptual model for this causal mediation analysis was illustrated in a directed acyclic graph (DAG) using DAGitty to identify confounding variables that need conditioning and adequate adjusting while addressing causal assumptions (S1 Fig) [52]. Apart from the potent confounders retrieved from a DAG, parenting style is still a possible confounding factor that needed to be adjusted in the model [9]. There are evidences showing parenting influences both screen use and behavioral problems [53, 54]. Therefore, we did another model analysis by adding the parenting factor as an additional covariate in the multivariable model to ensure the independent impact of excessive screen time on behavioral outcomes via EF (S2 Table and S2 Fig). We estimated the total effect (the average effect of screen time on behavior problems), average causal mediation effect (ACME; average screen time effect on the behavior problems through executive function), and average direct effect (ADE; average effect of screen time that impacted via all other mechanism excluding EF).

The sequential ignorability assumption was required for identifying ACME and ADE of the exposure [50, 51]. This assumption imposes two ignorability assumptions sequentially. First, the exposure is independent of the potential mediator and outcome, given the pre-exposure covariates. Second, the observed mediator is independent of the potential outcome, given the actual exposure and pre-exposure covariates [50]. Because the sequential ignorability assumption cannot be directly investigated with the observed data by statistical test, we performed a sensitivity analysis to quantify how robust the findings are to the violation of the sequential ignorability assumption [51].

### Statistical analysis

The statistical analyses were conducted using Stata version 16 (StataCorp, College Station, Texas, USA) [55]. The descriptive data was presented in mean (SD) and number (percentage) with the percentage of the missing data. Demographic and relevant information analyses were performed using the student t-test and Fisher’s exact test for continuous and categorical data, respectively. Multivariable Gaussian regression was analyzed to compare the BRIEF-P and SDQ scores between the excessive screen time and appropriate screen time groups, controlling for age, sex, socioeconomic status, and maternal education.

To support the mediation hypothesis, firstly, we conducted a preliminary regression analysis and found that there are statistically significant associations (p<0.05) between the exposure (screen time), mediator (executive function), and outcomes (behavioral problems). To identify the ACME and ADE of the association between excessive screen time and behavioral problems mediated by executive function, we performed a causal mediation analysis using the *medeff* command. This mediation analysis model was comprised of the exposure variable (T; screen time measured by the total duration of screen exposure), outcome variable (Y; behavioral problems measured by SDQ total difficulties score), and mediator (M; executive function measured by BRIEF-P global executive composite score), controlling for the potential confounders (x; sex, age, socioeconomic status, and maternal education). The exposure, mediator, and outcome were entered into the model as continuous variables. We used a bootstrap of 5,000 samples to handle the 95% CI for the mediating effect. The mediating effect with the 95% CI which did not include zero, was determined to be significant mediation.

To assess the robustness of the findings, we tested for violating the assumption of sequential ignorability via sensitivity analysis using *medsens* command. The sensitivity analysis result was displayed as the correlation parameter (Rho; ρ) between the residual variances of the models for the mediator and outcome. This parameter reflects the magnitude of mediator-outcome confounding required to vanish the mediating effect (ACME to be zero). The ACME estimate is more robust as this parameter increases. A pairwise deletion technique was done to deal with the missing data. A p-value less than 0.05 was considered statistically significant.

## Results

### Sample characteristics

**Fig 1:**
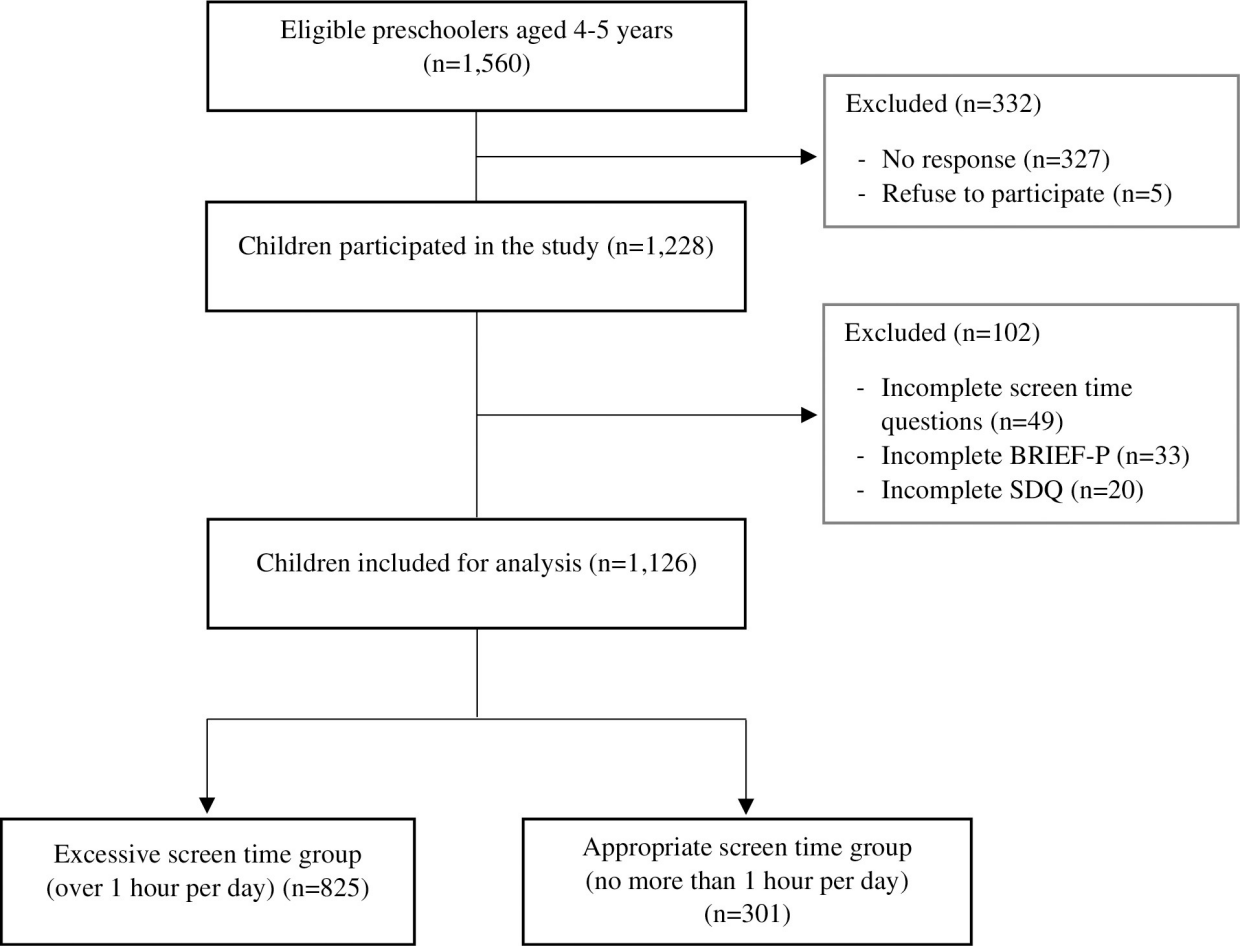
Study flow diagram.

A total of 1,126 typically developing participants aged four to five years (mean = 5.02 and SD = 0.56) were included in the analyses, as shown in the study flow diagram (Fig 1). Sample characteristics of excessive and appropriate screen time groups are presented in Table 1. There are no significant differences between the two groups except for sex. A majority of the study participants were preschoolers raised in middle-SES families with responsive parenting and whose mothers have at least a bachelor’s degree. Most of the children were also excessively exposed to the screen (over one hour per day, based on AAP recommendation [1]) and had an inadequate amount of moderate to vigorous physical activity (less than one hour per day followed by the age-appropriate guideline [45]).

**Table 1:** Baseline characteristics of excessive and appropriate screen time groups.

	Excessive screen time		Appropriate screen time		P-value	Missing
	(n = 825)		(n = 301)			n (%)
	mean	SD	mean	SD		
Male, n (%)	404	48.97	116	38.54	0.002	0
Age at assessment (years)	5.04	0.56	4.97	0.56	0.046	0
Socioeconomic status, n (%)						
Low	122	15.48	51	17.89	0.623	53 (4.71)
Middle	586	74.37	207	72.63		
High	80	10.15	27	9.47		
Maternal education, n (%)						
Below bachelor’s degree	173	21.65	74	25.43	0.191	36 (3.20)
Bachelor’s degree and above	626	78.35	217	74.57		
Parenting styles, n (%)						
Authoritative	796	96.84	298	99.00	0.112	3 (0.27)
Authoritarian	5	0.61	1	0.33		
Permissive	21	2.55	2	0.66		
Total screen time per day (hour)	2.18	1.02	0.68	0.32	<0.001	0
Total moderate to vigorous physical activity per day (hour)	0.56	0.42	0.55	0.38	0.925	2 (0.18)
Total sleep time (including naps) per day (hour)	10.80	1.18	10.95	1.18	0.066	4 (0.36)

SD, standard deviation.

### Comparing executive functions and behavioral problems between excessive and appropriate screen time groups

After controlling for age, sex, socioeconomic status, and maternal education, the excessive screen time group had higher BRIEF-P T-scores than the appropriate screen time group in all scales. The mean difference of T-scores between the two groups in the scales of shift, working memory, and GEC reached statistical significance (e.g., mean difference of GEC T-score = 1.49, 95% CI [0.12, 2.86], and *p* = 0.033) (Table 2). Since the higher T-scores reflect more difficulties in executive functioning, the excessive screen time group was likely to have more EF difficulties than the appropriate screen time group.

**Table 2:** The parent rated BRIEF-P T-scores and SDQ scores in excessive and appropriate screen time groups.

	Excessive screen		Appropriate screen		Mean difference	95% CI	P-value
	time (n = 825)		time (n = 301)				
	mean	SD	mean	SD			
**BRIEF-P T-score** ^**a**^							
Inhibit	51.53	8.88	50.36	8.96	1.17	-.013–2.36	0.052
Shift	47.75	7.55	46.40	7.62	1.35	0.34–2.36	0.009
Emotional control	47.00	9.29	46.18	9.37	0.82	-0.41–2.06	0.192
Working memory	55.87	11.00	54.04	11.10	1.83	0.36–3.29	0.015
Plan/organization	52.34	9.40	51.59	9.48	0.74	-0.51–2.00	0.245
Global executive composite	51.79	10.26	50.30	10.35	1.49	0.12–2.86	0.033
Clinically significant impaired EF (GEC score >/ = 65), n (%)	116 (14.06)		27 (8.97)		-	-	0.026
**SDQ score** ^**a**^							
Hyperactivity/inattention	3.98	2.14	3.59	2.16	0.40	0.11–0.68	0.007
Emotional problems	1.64	1.58	1.48	1.59	0.16	-0.05–0.37	0.132
Conduct problems	1.96	1.37	1.74	1.38	0.22	0.03–0.40	0.020
Peer problems	2.78	1.50	2.66	1.51	0.12	-0.08–0.32	0.224
Prosocial behavior	7.46	1.79	7.64	1.81	-0.18	-0.42–0.06	0.138
Total difficulties	10.37	4.55	9.47	4.59	0.90	0.29–1.50	0.004
Borderline and clinically significant behavior problems, n (%)	102 (12.36)		24 (7.97)		-	-	0.042

BRIEF-P, Behavior Rating Inventory for Executive Function–Preschool; SDQ, Strengths and Difficulties Questionnaire; EF, executive function; GEC, global executive composite; SD, standard deviation.

^a^Gaussian regression adjusted for age, sex, socioeconomic status, and maternal education.

When categorizing EF into impaired EF and normal EF status using a cut-off point (GEC T-score equal to or higher than 65 indicates clinically significant impaired EF), the excessive screen exposure group showed a significantly higher percentage of participants with impaired EF than the appropriate screen time group (Table 2).

For behavior problem outcomes, the excessive screen time group had higher SDQ scores on the difficulties scales and lower scores on the strengths scale than the appropriate screen time group. When considering the scales of difficulties, the mean difference of SDQ scores between the two groups in the scales of hyperactivity/inattention, conduct problems, and total difficulties reached statistical significance (e.g., mean difference of total difficulties score = 0.90, 95% CI [0.29, 1.50], and *p* = 0.004).

When categorizing behavior issues into borderline/clinically significant behavior problems and normal range behavior status using a cut-off point of SDQ total difficulties score [48], there was a significant difference in the number of children with borderline/clinically significant behavioral problems between the two groups. Specifically, the excessive screen time group had 4.4% more children who fell into this category than the appropriate screen time group (Table 2).

### Causal mediation analyses

**Fig 2:**
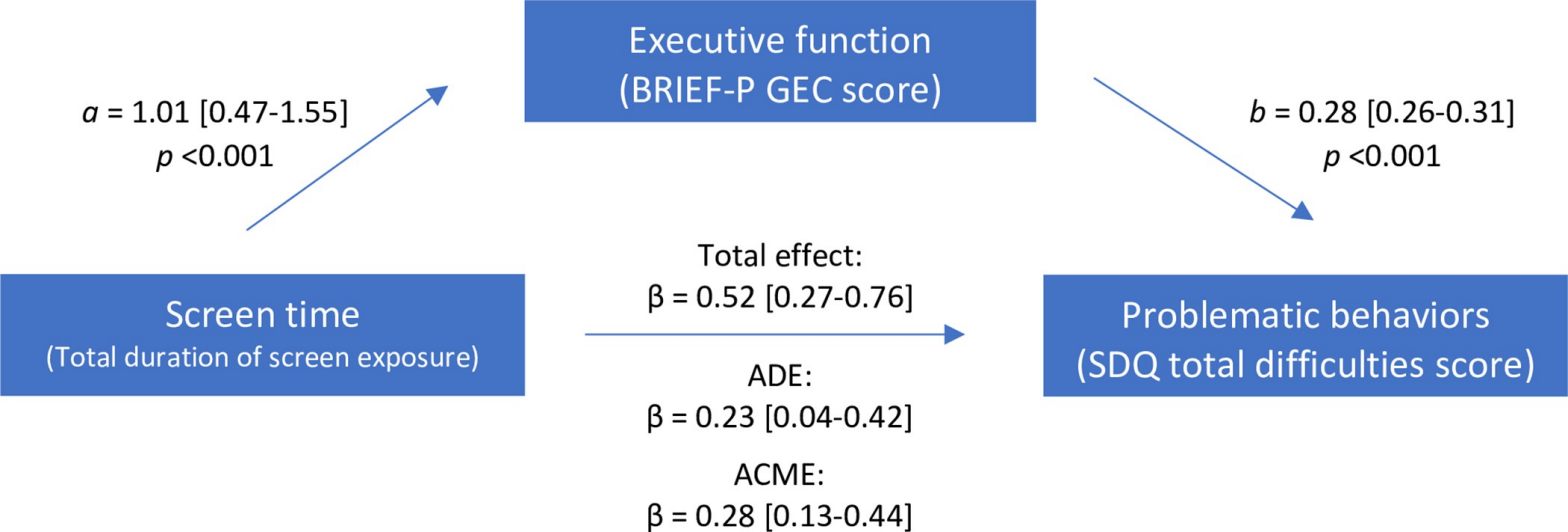
Causal mediation analysis. Causal mediation analysis shows effect estimates with a 95% confidence interval of the ADE and ACME between total screen time and the SDQ total difficulties score mediated by the BRIEF-P global executive composite score, adjusted for age, sex, socioeconomic status, and maternal education.

The results of the causal mediation analysis were presented in Table 3. After controlling for age, sex, socioeconomic status, and maternal education, the total effect of screen time on the SDQ total difficulties score was *β* = 0.52, 95%CI [0.27–0.76]. The ADE of such association was β = 0.23, 95%CI [0.04–0.42]. The ACME of screen time on behavioral problems mediated by executive functioning was β = 0.28, 95% CI [0.13–0.44] (Fig 2). More than half of the total effect was mediated through executive functioning (54.9%, 95%CI 37.4–100%). As previously mentioned, parenting style was one of the potential confounding factors of this relationship. The same model with additional adjusting for parenting style was conducted, and the result showed a similar trend to the main finding (S2 Table and S2 Fig).

**Table 3:** Estimated mediation analysis examining the mediated effect of executive function on the association between screen time and problematic behaviors using *medeff*^a^.

Equation	Description	Effect parameter	β	95% CI	P-value
m = *f* (x)	Effect of screen time on EF	a	1.01	(0.47, 1.55)	<0.001
Y = *f* (m)	Effect of EF on behavioral problems	b	0.28	(0.26, 0.31)	<0.001
Y = *f* (x)	Effect of screen time on behavioral problems	c	0.52	(0.28, 0.76)	<0.001
Y = *f* (m x)	Effect of EF on behavioral problems adjusted by screen time	b’	0.28	(0.26, 0.30)	<0.001
	Effect of screen time on behavioral problems adjusted by EF	c’	0.23	(0.05, 0.42)	0.014
	Total effect		0.52	(0.27, 0.76)	<0.001
	Average direct effect (ADE)		0.23	(0.04, 0.42)	0.014
	Average causal mediated effect (ACME)		0.28	(0.13, 0.44)	
	Proportion mediated (%)		54.9	(37.4, 100)	
**Sensitivity results**					
	Rho at which ACME = 0		0.63		

β, beta-coefficient; CI, confidence interval; EF, executive function.

^a^The model was adjusted for age, sex, socioeconomic status, and maternal education.

**Fig 3:**
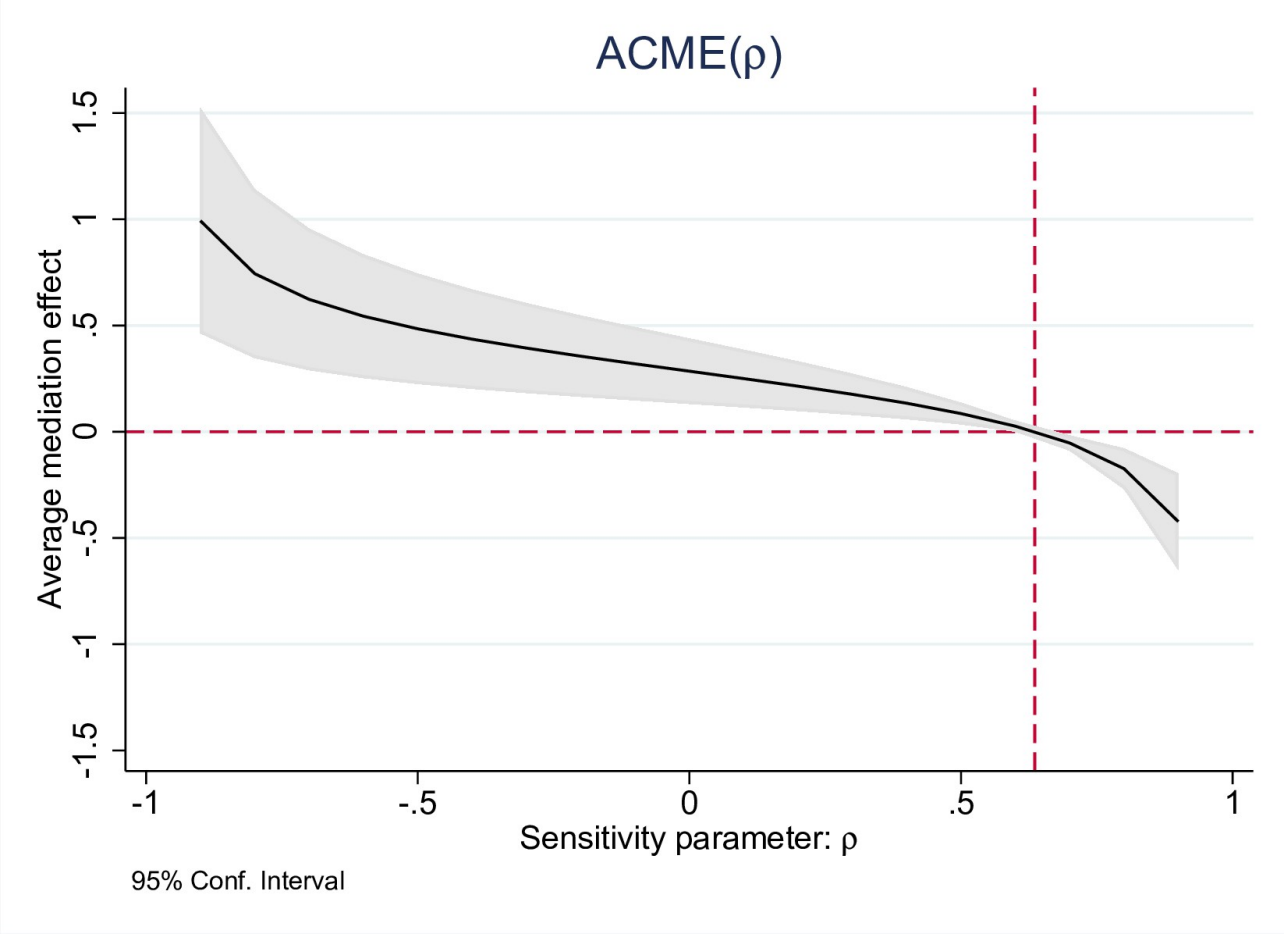
Sensitivity analysis. Average causal mediation effect as a function of the degree of violation of sequential ignorability assumption (sensitivity parameter: ρ).

The sensitivity analysis revealed that the value for ρ at which ACME = 0 was 0.63 (Fig 3). This suggested that a moderate amount of confounding was required to result in no ACME. Thus, the estimated ACME of executive function on the association between screen time and behavioral problems was fairly robust to the sequential ignorability violation.

## Discussion

The proposed conceptual model used in this study, when adjusted for age, sex, socioeconomic status, and maternal education, indicated that children with excessive screen time were more likely to have EF difficulties and behavioral problems than children with appropriate screen use. Furthermore, excessive screen time had direct and mediating effects on behavioral problems. The increased behavioral problems in preschoolers exposed to excessive media might be partially explained by their impaired executive functioning.

The direct effect of media use on behavioral problems was significant in this study. Children with excessive screen exposure were likely to have more problematic behaviors, particularly externalizing behaviors (i.e., hyperactivity and conduct problems). The results of this study were consistent with prior research [37, 56, 57]. There was a five times greater risk to have externalizing problems in children with screen time greater than 2 hours per day compared to children with less than 30 minutes per day [56]. Meta-analyses concluded that there was a small significant association between screen time and behavioral problems, especially ADHD-related behaviors, in children and adolescents [37, 57]. Differential Susceptibility to Media effects Model (DSMM), a theoretical model explaining media effects on behavioral problems, posits the response states mechanism underlying such a relationship [58]. Habituation from repeated exposure to rapid conversion of screen pictures can reduce the arousal threshold, and ultimately lead to hyperactivity symptoms. As for contributing to attention deficits, children find it difficult to engage in tasks that demand sustained concentration because the fast-pacing screen drives them to often switch their attentions [12, 58]. Although other studies have commonly shown co-occurring increased externalizing and internalizing behaviors [59], this study did not show significant differences in internalizing behaviors (i.e., emotional and peer problems) across the groups. The result was in contrast to prior studies [37, 60]. The rationale for the insignificant difference was that, in comparison to internalizing behaviors, externalizing ones are more obviously noticed by parents and teachers, especially in young children. It is warranted to conduct further research on the mechanisms that underlie screen time’s effects on externalizing versus internalizing behaviors. Screen effects on externalizing behaviors may be explained by violent media, whereas internalizing issues may be due to social isolation and sleep disturbances [37].

This study proved a significant ACME of EF that partially mediated excessive screen time and behavioral problems. Children who spend much time watching screens tend to experience executive dysfunction. Consequently, impaired EF may result in difficulties in self-regulation, emotional control, and social coping, ultimately leading to behavioral issues [61]. Growing evidence has shown that the association between inappropriate screen use and behavioral problems may be explained by functional and structural brain alterations. For instance, exposure to violent content on television could temporarily activate a network of brain areas that are related to emotion regulations, attention, and arousal levels [62]. Additionally, a prior study demonstrated a link between excessive screen time and ADHD through altered white matter connectivity [63]. These brain alterations were possibly associated with EF deficits, as explained by the prior study that the increased usage of screen-based media links to the microstructural changes of both grey matter (decreased in cortical thickness and sulcal depth) and white matter (lower integrity) in brain regions supporting multiple skills, including executive functioning [64, 65]. Furthermore, a previous study has shown that the cortical electroencephalogram activity in the frontal and parietal regions mediated the link between screen exposure in infancy and later worse EF outcomes in school ages [25]. These frontoparietal regions involve several EF-specific skills, including working memory and shifting [66, 67]. Apart from the biological mechanisms, the plausible explanations in the aspects of social/behavioral mechanisms of excessive screen time on EF depletion are that screen time reduces parent-child interactions, real-world social connections, and time engaging in physical activities, all of which are critical foundations in fostering EF [18, 68]. Alternatively, screen overuse affects a child’s language development [69]. Since language facilitates processing information and representational thought via verbal labels, it was essential for supporting early EF development in preschool ages [70, 71].

To our knowledge, this is the first study using a mediation analysis to comprehend the role of executive functioning in the relationship between excessive screen usage and behavioral issues in preschool children. Although the mean differences in EF and SDQ difficulties scores between excessive and appropriate media groups were small, the excessive media exposure group exhibited a larger proportion of participants with EF deficits and at-risk behavioral problems compared to appropriate media group. There was a trend suggesting that excessive screen time might contribute to EF and behavioral issues in children. Since there is a critical window for EF development, early EF-targeted promotions and interventions should be provided to at-risk children before the EF brain circuits fully mature. Although many biological and environmental factors contribute to EF outcomes [39], media exposure is a more manageable environmental factor influencing EF development. Parents, teachers, and health care providers should monitor screen use in children to prevent their children from having later adverse outcomes. However, it is important to use caution when interpreting the findings of this study because the sensitivity analysis revealed an unmeasured confounder with a positive correlation between the mediator and the outcome. Moreover, EF might not be the sole mediator to explain such an effect thoroughly. Other potential mediating factors, which might be both consequence of screen exposure and a precursor of a young child’s behaviors, such as physical activity level, sleep pattern, and excess weight status [72, 73], were needed to elucidate the relationship in future research. It is also necessary to conduct longitudinal studies and replicate the results of this investigation with additional consideration of moderators and cross-cultural effects existing in the association.

There are limitations to this study. First, we cannot explicitly conclude the causation and direction of the association because of the cross-sectional study design. The direction of the association could be excessive screen time leading to behavioral problems. Conversely, it is also possible that children exhibiting problematic behaviors might experience increased screen time due to their parents’ perception of screens as an effective way to calm their children down [60, 74]. Furthermore, parenting stress resulting from dealing with the child’s challenging behaviors creates the tendency to permit more screen time as a coping mechanism. This is done in an effort to manage their own parenting stress and avoid triggering further parental stress brought on by imposing limits on their child’s screen time [74, 75]. Alternatively, there could be a reciprocal relationship between excessive screen time and behavior dysregulation, with each one reinforcing the other [60]. Secondly, the questions on screen time in this study were based on parents/guardians’ estimation and not an objective measure. Although monitoring devices and screen time logbooks are the most accurate objective measurements, these methods take much time and effort to measure. To reduce parents’ burden, we used survey questions regarding screen time and encouraged the main caregiver, who spends the most time with the child and monitors the child’s screen use during the day, to answer these specific questions. Thirdly, other aspects of screen use, such as types (e.g., foreground vs. background), contents (e.g., violent vs. nonviolent), contexts (co-viewing vs. watching alone), and the onset of use (early onset vs. late onset), were not collected in this study. These aspects of screen use might also influence EF and behaviors and could be essential to obtaining a comprehensive conclusion regarding screen use and behavioral problems. Fourthly, other characteristics, such as the child’s intellectual quotient and temperament, were not controlled in the study. These factors might confound the association between excessive screen time and behavior problems. Finally, data included in the analysis was based solely on the parent reports, which may generate a reporting bias. However, BRIEF-P and SDQ were standardized measurements with good reliability and validity [46, 49]. There were acceptable inter-rater agreement correlations between parent and teacher reports of BRIEF-P and SDQ questionnaires [46, 49].

In conclusion, excessive screen time may have a direct impact on behavioral problems in preschoolers. Executive dysfunction seems to mediate the effect of excessive screen time on behavioral issues, as this study shows that more than half of the total effect was mediated through executive function deficits. However, the findings from this study need to be interpreted with caution due to the existence of residual unmeasured confounders presented by sensitivity analysis. Moreover, there might be a generalizability limitation due to the study’s samples being restricted to the Thai population. The results may raise concerns about the importance of monitoring screen time, executive function deficits, and behavioral problems among young children to activate prompt interventions. Further research is warranted to investigate the moderating effects influencing this relationship and EF-targeted interventions to reduce problematic behaviors in young children with high exposure to media.

## Supporting information

S1 Fig Directed acyclic graph (DAG). DAG presents the conceptual model for the mediation analysis. Four confounders (ancestor of exposure and outcome) were determined as the minimal sufficient adjustment sets for estimating the average direct and causal mediation effects of screen time on behavioral problems.
(TIF)

S2 Fig Mediation analysis. Mediation analysis shows effect estimates with a 95% confidence interval of the ADE and ACME between total screen time and the SDQ total difficulties score mediated by the BRIEF-P global executive composite score, adjusted for age, sex, socioeconomic status, maternal education, and parenting style.
(TIF)

S1 Table Inter-rater reliability and instrumental validity of physical activity and sleep questions. (PDF)

S2 Table Estimated mediation analysis examining the mediated effect of executive function on the association between screen time and problematic behaviors using *medeff*. (PDF)

## Abbreviations

AAP: American Academy of Pediatrics
ACME: average causal mediation effect
ADE: average direct effect
BRIEF-P: Behavior Rating Inventory of Executive Function-Preschool
EF: executive function
GEC: global executive composite
PSDQ: Parenting Styles and Dimensions Questionnaires
SDQ: Strengths and Difficulties Questionnaire
SES: socioeconomic status
