# Supplementary figures and images for: Executive dysfunction as a possible mediator for the association between excessive screen time and problematic behaviors in preschoolers
Source: PLoS One. 2024 Apr 4;19(4):e0298189. doi: 10.1371/journal.pone.0298189 (PMC10994291; doi:10.1371/journal.pone.0298189)

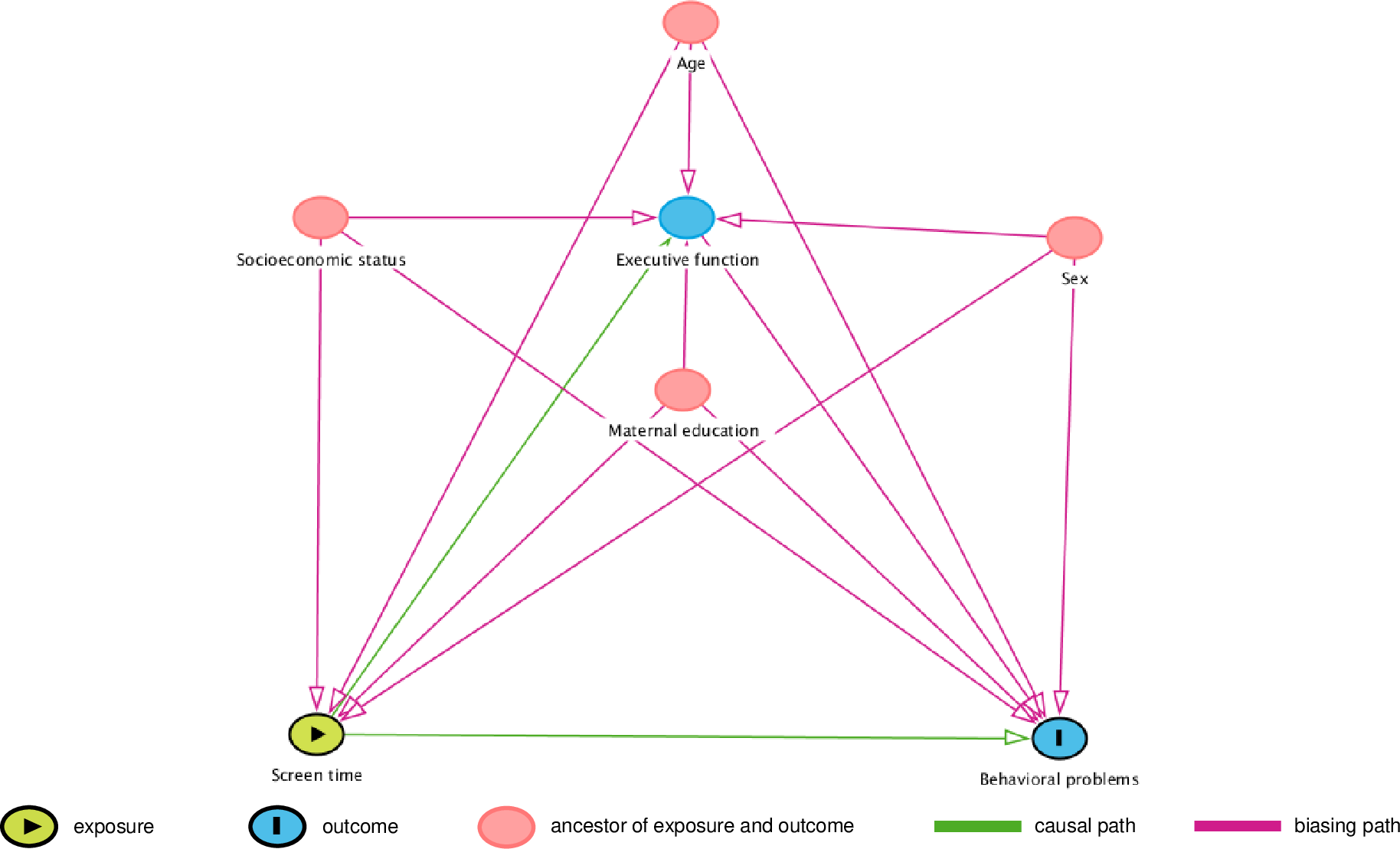

Supplement: S1 Fig — DAG presents the conceptual model for the mediation analysis. Four confounders (ancestor of exposure and outcome) were determined as the minimal sufficient adjustment sets for estimating the average direct and causal mediation effects of screen time on behavioral problems. (TIF) [file pone.0298189.s001.tif]

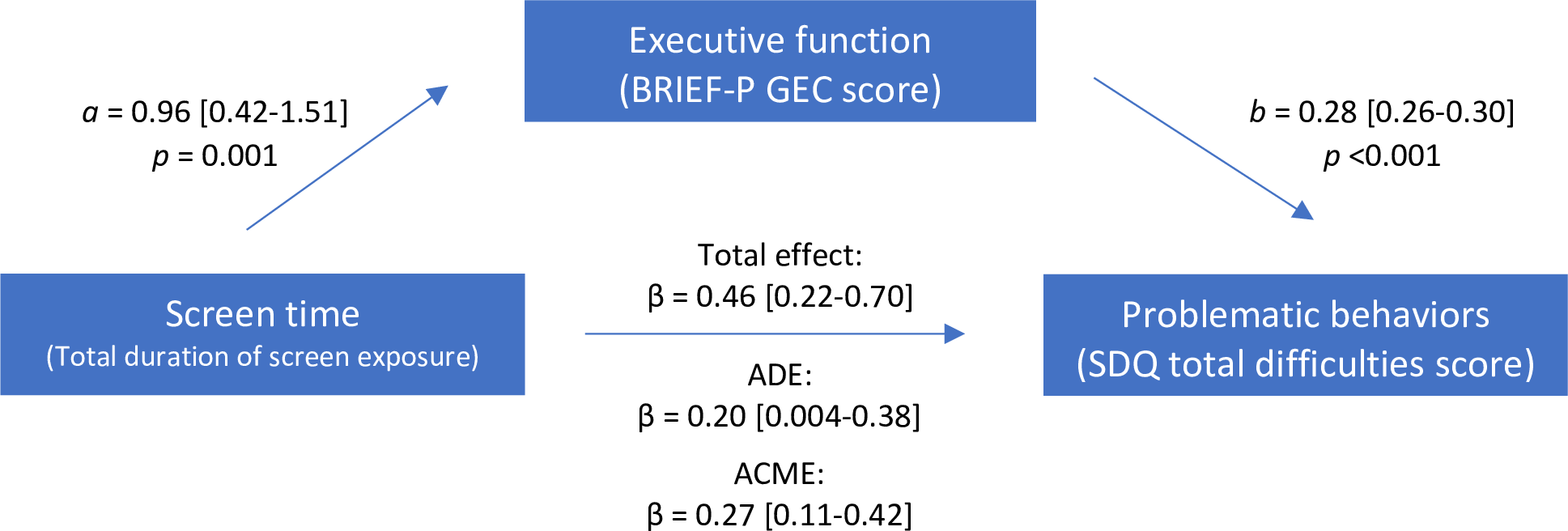

Supplement: S2 Fig — Mediation analysis shows effect estimates with a 95% confidence interval of the ADE and ACME between total screen time and the SDQ total difficulties score mediated by the BRIEF-P global executive composite score, adjusted for age, sex, socioeconomic status, maternal education, and parenting style. (TIF) [file pone.0298189.s002.tif]
