# Supplementary material for: Executive dysfunction as a possible mediator for the association between excessive screen time and problematic behaviors in preschoolers
Source: PLoS One. 2024 Apr 4;19(4):e0298189. doi: 10.1371/journal.pone.0298189 (PMC10994291; doi:10.1371/journal.pone.0298189)
Supplement: S1 Table — (PDF) [file pone.0298189.s003.pdf]

Supplementary Table 1.

Inter-rater reliability and instrumental validity of physical activity and sleep questions.

Questions	Inter-rater reliability[a]	Instrumental validity[b]
Physical activity	0.74	0.62
Sleep	0.78	0.62

[a]The inter-rater reliability was tested in 10 mother-father pairs who reported on their child’s daily physical activities and sleep. A physical activity question was given to each parent separately, asking them to rate how often per week and how long per day their child engaged in active play on weekdays and weekends. The average daily duration of moderate to vigorous physical activity (MVPA) was calculated to categorize into adequate (at least 60 minutes/day) or inadequate exposure to MVPA (less than 60 minutes/day). The agreement of the child's MVPA exposure was evaluated by comparing the reports from the mother and father using Cohen's kappa coefficient analysis. The same MVPA evaluation method was used to assess the inter-rater reliability of the sleep questions.

[b]Instrumental validity was tested in 10 preschooler-mother dyads. The child agreed to wear a heart rate monitoring device (smart band), and the child’s parent was given informed consent. For one week, heart rate was tracked to get factual information. MVPA duration accumulated per day was extracted from the wearable device by searching for the time when the heart rate spiked. In order to confirm that the increased heart rate was a result from MVPA, these data were corroborated by the record book, in which the mother noted the day and time the child participated in energetic activities. The amount of MVPA the child performed on average each day was calculated and classified as either adequate or inadequate exposure to MVPA. To examine the validity of the MVPA questions, the data of a parent’s report and the smart band were computed using Cohen's kappa coefficient analysis. The validity of the sleep question was also tested using the same method of MVPA.
