# Supplementary material for: Executive dysfunction as a possible mediator for the association between excessive screen time and problematic behaviors in preschoolers
Source: PLoS One. 2024 Apr 4;19(4):e0298189. doi: 10.1371/journal.pone.0298189 (PMC10994291; doi:10.1371/journal.pone.0298189)
Supplement: S2 Table — (PDF) [file pone.0298189.s004.pdf]

Supplementary Table 2.

Estimated mediation analysis examining the mediated effect of executive function on the association between screen time and problematic behaviors using *medeff*.[a]

Equation	Description	Effect parameter	β	95% CI	P-value
m=*f* (x)	Effect of screen time on EF	a	0.96	(0.42, 1.51)	0.001
Y=*f* (m)	Effect of EF on behavioral problems	b	0.28	(0.26, 0.30)	<0.001
Y=*f* (x)	Effect of screen time on behavioral problems	c	0.46	(0.22, 0.71)	<0.001
Y=*f* (m x)	Effect of EF on behavioral problems adjusted by screen time	b'	0.28	(0.26, 0.30)	<0.001
	Effect of screen time on behavioral problems adjusted by EF	c'	0.20	(0.01, 0.38)	0.041
	Total effect		0.46	(0.22, 0.70)	<0.001
	Average direct effect (ADE)		0.20	(0.004, 0.38)	0.041
	Average causal mediated effect (ACME)		0.27	(0.11, 0.42)	
	Proportion mediated (%)		57.3	(38.2, 100)	
**Sensitivity results**					
	Rho at which ACME=0		0.63		

β, beta-coefficient; CI, confidence interval; EF, executive function.

[a]The model was adjusted for age, sex, socioeconomic status, maternal education, and parenting style.
